# Short-Term Psychophysiological Effects of Immersive Versus Non-Immersive Relaxation in University Students: A Randomized Controlled Trial

**DOI:** 10.3390/healthcare14152322

**Published:** 2026-08-01

**Authors:** Aleksandra Nowakowska, Magdalena Nowak, Jacek Bigas, Zofia Gaweł, Mirosław Nęcki, Sebastian Rutkowski

**Affiliations:** 1 Doctoral School, Opole University of Technology, 45-061 Opole, Poland; 2 Descartes’ Error Student Research Association, Department of Physical Education and Physiotherapy, Opole University of Technology, 45-758 Opole, Poland; jacek.bigas@student.po.edu.pl (J.B.); zofia.gawel@student.po.edu.pl (Z.G.); 3 Department of Interstitial Lung Diseases and Transplantology, John Paul II Hospital, 31-202 Kraków, Poland; mireknec@poczta.onet.pl; 4 Department of Physical Education and Physiotherapy, Opole University of Technology, 45-758 Opole, Poland

**Keywords:** virtual reality, immersive relaxation, heart rate variability, perceived stress, university students, autonomic nervous system, cybersickness, digital mental health, stress reduction

## Abstract

**Background:** University students are particularly vulnerable to elevated stress, anxiety, and reduced psychological well-being, especially in the post-pandemic period. Immersive virtual reality (VR) has emerged as a promising technology-supported approach for relaxation and stress reduction; however, evidence regarding its short-term psychophysiological effects in academic populations remains limited. **Objective:** The aim of this study was to evaluate the effects of a short-term immersive VR relaxation intervention on perceived stress and autonomic nervous system activity in university students. **Methods:** A randomized controlled study with repeated measures was conducted among university students from Opole, Poland. Participants were allocated to an immersive VR group or a non-immersive screen-based control group. Both groups received identical relaxation content for 10 min daily over five consecutive days, differing only in the level of immersion. Heart rate variability (HRV) was recorded continuously during four consecutive 5-minute epochs within each session. Perceived stress was assessed using the Perceived Stress Scale-10 before and after the intervention. Cybersickness symptoms were assessed in the VR group. **Results:** Immersive VR elicited more pronounced favourable changes in HRV parameters than non-immersive exposure, including increased lnRMSSD, SDNN, and PNS index values, together with reduced mean heart rate, SNS index, and Stress index. Both groups showed significant reductions in perceived stress; however, the reduction was significantly greater in the VR group (*p* = 0.009, Cohen’s d = 0.78). Cybersickness symptoms were low and decreased across the intervention period. **Conclusions:** Short-term immersive VR relaxation may represent a feasible and well-tolerated approach for reducing perceived stress and supporting autonomic regulation in university students under controlled experimental conditions. However, the findings should be interpreted with caution due to the short intervention period and the absence of follow-up. Furthermore, adequately powered randomized controlled trials with longer observation periods are required to confirm these preliminary findings.

## 1. Introduction

In recent years, there has been a marked increase in interest in the mental health of university students, largely driven by the rising prevalence of stress- and anxiety-related symptoms in this population. The period of university education, which coincides with early adulthood, represents a critical developmental stage characterized by profound psychological, social, and vocational transitions. These changes often occur alongside substantial academic demands. The literature emphasizes that university students constitute a population particularly vulnerable to chronic stress, resulting from the cumulative impact of diverse stressors, including performance pressure, excessive academic workload, academic competition, uncertainty regarding future career prospects, and the need to adapt to new living conditions. Epidemiological studies indicate that a substantial proportion of students experience moderate-to-high levels of stress, as confirmed by both cross-sectional analyses and systematic reviews conducted across different countries and educational systems [1,2,3]. Importantly, elevated stress symptoms in this group frequently coexist with increased levels of anxiety and other emotional difficulties, highlighting the need to consider student mental health as a key area of public health intervention.

The burden of stress- and anxiety-related problems among university students was further exacerbated by the global social changes triggered by the COVID-19 pandemic. The introduction of sanitary restrictions, social isolation, and the abrupt transition to remote learning substantially altered the functioning of the academic environment, increasing uncertainty and psychological burden among students. Evidence from meta-analyses and multicentre studies indicates that the prevalence of depressive and anxiety symptoms in this population increased significantly following the outbreak of the pandemic. A meta-analysis including university students from various countries showed that, during the pandemic, depressive symptoms were present in an average of 39% of students, whereas anxiety symptoms were observed in approximately 36% of participants, representing a clear increase compared with the pre-pandemic period [4]. In addition, studies conducted among university students in the United States showed that as many as 71% of respondents reported an increase in stress and anxiety during the pandemic, identifying social isolation, health-related concerns, and difficulties associated with remote learning as the main stressors [5]. Particularly relevant are findings from studies conducted among students in Poland during the COVID-19 pandemic, which indicate a substantial exacerbation of mental health problems in this population. It was shown that 58% of students presented elevated levels of stress, while depressive symptoms were observed in 56% of participants. Moreover, 18% of respondents reported suicidal ideation, indicating the serious nature of the psychological consequences associated with functioning under conditions of social isolation and remote education. These findings highlight the scale of the problem and suggest that chronic stress in the student population may contribute not only to reduced psychological well-being, but also to the development of severe emotional disorders requiring specialist intervention [6].

The increasing burden of stress- and anxiety-related problems among university students highlights the need to identify effective, accessible, and acceptable interventions aimed at improving mental health. Conventional approaches, including psychotherapy, relaxation training, mindfulness-based techniques, and physical activity, may reduce emotional tension, but their use in student populations is often limited by access barriers, lack of time, low motivation, and stigma associated with seeking psychological support [7]. Therefore, technology-supported interventions that are brief, engaging, and feasible for academic settings have gained increasing attention [8]. Particular interest has focused on immersive technologies, which, by promoting high levels of user engagement and enabling the creation of controlled conditions for psychological intervention, may represent a promising tool for reducing stress and anxiety [9,10].

In response to the growing need for innovative approaches to support mental health, the use of immersive virtual reality (VR) in interventions aimed at reducing stress and anxiety has attracted increasing attention. VR technology enables the creation of fully controlled, multisensory environments that elicit a sense of presence in users, thereby facilitating deep emotional and cognitive engagement [11,12]. The literature suggests that such immersive experiences may effectively modulate stress responses by redirecting attention away from stress-inducing stimuli, inducing a state of relaxation, and influencing autonomic nervous system activity. Previous studies have shown that relaxation interventions delivered in VR environments may lead to significant reductions in stress and anxiety levels in both clinical and non-clinical populations. Experimental studies have demonstrated that exposure to immersive natural environments, such as forest or coastal landscapes, is associated with reduced emotional tension and improved subjective psychological well-being [13,14]. Moreover, meta-analyses suggest that VR-based interventions may be as effective as, and in some cases more engaging than, conventional relaxation techniques, thereby increasing their applicability in young-adult populations [15].

Despite the growing body of evidence supporting the effectiveness of VR-based interventions, important knowledge gaps remain regarding the optimal parameters of their application, including session duration, the type of immersive environment, and exposure frequency. In particular, there is still a limited number of studies focusing on short-term relaxation interventions delivered under conditions resembling students’ everyday functioning, which is of key importance from the perspective of implementing such solutions in academic settings. Furthermore, existing reports indicate considerable heterogeneity in the research protocols used, making it difficult to draw definitive conclusions regarding the effectiveness of VR as a tool for reducing stress and anxiety. Another important aspect requiring further exploration is the impact of immersive relaxation environments on different components of the stress response, including both subjective psychological experiences and potential changes in physiological functioning. Accordingly, experimental studies using standardized measurement tools are increasingly needed to allow for a comprehensive evaluation of intervention effectiveness.

In the student population, it is particularly important to develop solutions that are not only effective but also easy to implement, attractive to users, and feasible for use in university settings without the need for extensive therapeutic infrastructure. Consequently, modern digital interventions, including immersive VR-based approaches, are increasingly investigated as potential methods for supporting psychological well-being and stress regulation.

In response to these needs, the aim of the present study was to evaluate the effects of a short-term relaxation intervention delivered using immersive virtual reality on perceived stress and autonomic nervous system activity in university students. Specifically, this study examined whether immersive delivery of identical relaxation content would produce greater short-term changes in perceived stress and heart rate variability than non-immersive screen-based delivery.

## 2. Materials and Methods

**Figure 1:**
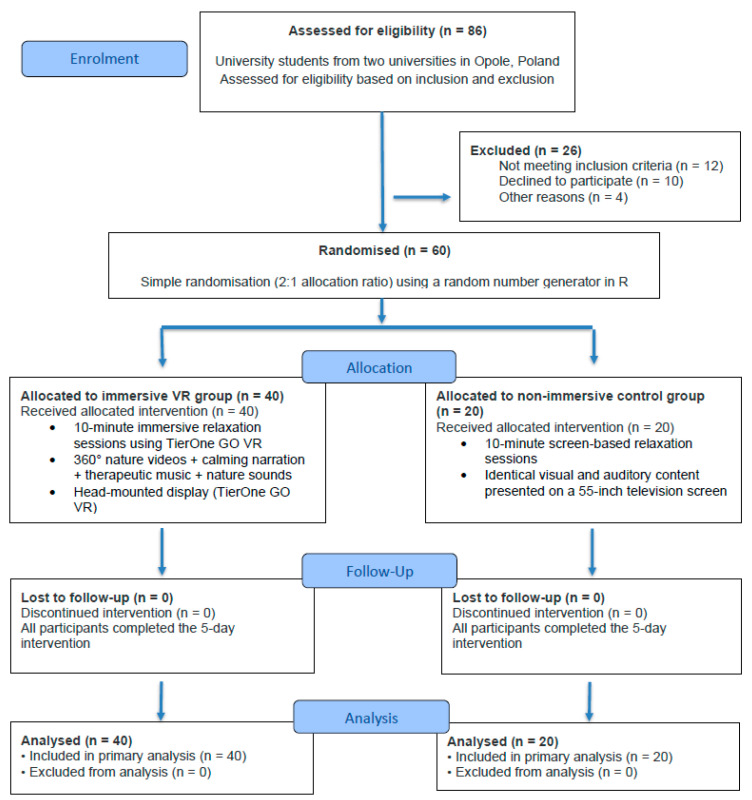
CONSORT 2025 Flow Diagram [16], this is an Open Access article distributed under the terms of the Creative Commons Attribution License (https://creativecommons.org/licenses/by/4.0/ (accessed on 23 July 2026)), which permits unrestricted use, distribution, and reproduction in any medium, provided the original work is properly.

This study was designed as a randomized controlled trial with repeated measures to assess the effects of a short-term immersive virtual reality relaxation intervention on autonomic nervous system activity and perceived stress in university students. A total of 60 students from two universities in Opole, Poland, were included in the final analysis. Participants were randomly assigned to either the immersive VR group (*n* = 40) or the non-immersive screen-based control group (*n* = 20) using simple randomization with a predefined 2:1 allocation ratio. The random allocation sequence was generated before participant enrolment using the random number generator implemented in R statistical software. Allocation was performed only after confirmation of eligibility and provision of written informed consent. The allocation sequence was not available to the researcher responsible for final statistical analyses, and statistical analyses were performed using anonymized datasets after completion of data collection. No post-randomization subsampling, exclusion, or reallocation was performed. The 2:1 allocation ratio was chosen a priori because the primary physiological aim of this proof-of-concept study was to characterize short-term autonomic responses to immersive VR across repeated exposures, while retaining a smaller active comparator group exposed to the same relaxation content without immersion. This allocation strategy increased the amount of safety, tolerability, and HRV data collected under immersive VR conditions, while preserving a controlled comparison with non-immersive screen-based delivery of identical audiovisual content. No stratification procedure was applied because simple randomization was used. Owing to the nature of the intervention, participant blinding was not feasible. Outcome assessors were not blinded because all measurements were collected directly by the research team according to a standardized protocol. Statistical analyses were performed after completion of data collection using anonymized datasets; however, group labels were required for the planned between-group analyses. Group-specific demographic and clinical characteristics are presented in Table 1, and students represented various academic disciplines, predominantly physiotherapy and physical education. All participants were full-time undergraduate students. Baseline demographic and clinical characteristics were assessed to describe the study groups and are presented in Table 1. This study is reported in accordance with the CONSORT 2025 guidelines for randomized controlled trials. Participant enrolment, allocation, follow-up, and analysis are summarized in the CONSORT flow diagram (Figure 1).

### 2.1. Qualification Criteria

Students aged 18–30 years without contraindications to VR use (e.g., uncorrected visual impairment, balance disorders, neurological or psychiatric conditions, or concurrent psychological/pharmacological treatment) were eligible. Written informed consent was obtained from all participants, and this study was conducted in accordance with the Declaration of Helsinki. The protocol was approved by the Bioethics Committee of the Opole Chamber of Physicians (Resolution No. 288, 7 June 2019) and registered at ClinicalTrials.gov (NCT06480409).

### 2.2. Intervention

Participants were randomly assigned to one of two experimental conditions that differed only in the level of immersion while maintaining identical therapeutic content and duration. The immersive intervention was delivered using the TierOne GO virtual reality system (VR TierOne, Stanowice, Poland). Participants wore high-resolution head-mounted goggles that presented 360° nature videos accompanied by a calming narrated script, therapeutic music, and nature sounds delivered through integrated headphones. Each session lasted approximately 10 min and guided participants through diaphragmatic breathing, progressive muscle relaxation, and vivid guided imagery of peaceful environments.

In the non-immersive control condition, the same visual and auditory content was presented on a standard 55-inch television screen without head-mounted display or stereoscopic immersion. Both conditions were conducted daily for five consecutive days in a quiet, dimly lit room. Participants remained seated on a beanbag chair to ensure comfort and to minimise movement artefacts during physiological recordings.

The measurement protocol was identical in both groups and is illustrated in Figure 1. It consisted of four consecutive 5-minute epochs within each session: resting baseline (T1), first half of the relaxation content (T2), second half of the relaxation content (T3), and post-session rest (T4). Heart rate variability was recorded continuously throughout all epochs. Only one participant was present in the room at a time, with two members of the research team responsible for technical setup and monitoring outside the room. This design allowed for isolation of the specific contribution of immersive VR while controlling for all other procedural variables. Before each session, participants were instructed to silence their mobile phones and leave them in a designated basket outside the intervention area to minimize potential distractions. Participants in both groups were instructed to attend continuously to the presented audiovisual content throughout the entire session. Adherence to the intervention protocol was monitored by the research staff, and no interruptions or protocol deviations were observed during the intervention sessions.

### 2.3. Measurements

Cardiac signal recording was performed using a Polar H10 monitor (Polar Electro Oy, Kempele, Finland), which enables the measurement of RR intervals with high temporal resolution, with a sampling frequency of 1000 Hz. This device is widely recommended as a reference tool for assessing heart rate variability (HRV) under experimental conditions, providing high accuracy in the detection of electrocardiographic signals [17]. The recorded data were used to assess autonomic nervous system activity, which represents a key marker of physiological responses to relaxation interventions. HRV analysis was performed using dedicated Kubios HRV Premium software 3.5.0 (Kubios Oy, Kuopio, Finland), which enables advanced signal processing, including artefact correction and the precise calculation of time-domain, frequency-domain, and non-linear parameters. HRV acquisition, preprocessing, and reporting were conducted in accordance with current methodological recommendations for human heart rate and heart rate variability research [18]. The analysis included indices reflecting autonomic nervous system activity, with particular emphasis on the PNS index, which represents parasympathetic nervous system activation and is directly associated with regenerative processes and the physiological state of relaxation. The primary physiological outcome was lnRMSSD, selected as a widely used marker of vagally mediated parasympathetic cardiac modulation and short-term relaxation-related autonomic regulation. The PNS index was treated as a key secondary autonomic outcome, whereas mean HR, SDNN, SNS index, Stress index, and additional spectral HRV parameters were considered secondary or exploratory outcomes.

Perceived stress was assessed using the Polish version of the Perceived Stress Scale-10 (PSS-10). The PSS-10 is a self-report questionnaire consisting of 10 items designed to measure the degree to which participants perceive situations in their lives as stressful. Each item is rated on a 5-point Likert scale ranging from 0 to 4, where 0 indicates “never” and 4 indicates “very often”. Four positively worded items are reverse-scored before calculating the total score. The final score is obtained by summing all item scores and ranges from 0 to 40, with higher scores indicating a higher level of perceived stress [19]. In the present study, the PSS-10 was administered twice: before the beginning of the intervention on day 1 and after completion of the five-day intervention on day 5. The change in PSS-10 score between day 1 and day 5 was used to assess the effect of the intervention on perceived stress.

Cybersickness symptoms were assessed using the Virtual Reality Sickness Questionnaire (VRSQ). The VRSQ is used to evaluate adverse symptoms associated with exposure to virtual reality environments and includes subscales related to nausea, oculomotor discomfort, and disorientation. Higher scores indicate greater severity of cybersickness symptoms [20]. In the present study, cybersickness was assessed only in the immersive VR group, as participants in the control group were not exposed to a head-mounted virtual reality environment. VRSQ scores were collected across the five-day intervention period to evaluate the tolerability of repeated immersive VR exposure and to monitor changes in cybersickness symptoms over consecutive sessions.

### 2.4. Statistical Analysis

The normality of variable distributions was assessed using the Shapiro–Wilk test. Given the non-normal distribution of most HRV parameters (*p* < 0.05), natural-log transformation was applied where appropriate (e.g., lnRMSSD), in line with current recommendations for psychophysiological research [21]. For each HRV parameter, all available repeated measurements from the five intervention days and four within-session epochs were included in a single linear mixed-effect model. Thus, the data structure consisted of repeated 5-minute epoch-level observations nested within intervention days and participants. Fixed effects included Group, Time, the Group × Time interaction, and Day to account for repeated exposure across the five sessions. Participant ID was included as a random effect to account for within-participant correlation across repeated measurements. The primary effect of interest was the Group × Time interaction, reflecting whether within-session autonomic changes differed between immersive VR and non-immersive screen-based relaxation. Higher-order interactions involving Day were not included in the primary model because this study was primarily designed to evaluate short-term within-session psychophysiological responses rather than cumulative day-by-day training effects. Because a statistically significant baseline difference in age was observed between groups, age was considered as a potential confounding factor in the interpretation of the findings. The main HRV analyses were based on the predefined repeated-measures linear mixed-effect model focused on within-session Group × Time effects across the four experimental epochs. Baseline lnRMSSD values were nearly identical between groups, suggesting comparable parasympathetic cardiac modulation before the intervention. Nevertheless, the potential influence of baseline age imbalance was explicitly acknowledged in the Limitations section. Model assumptions (normality and homoscedasticity of residuals, absence of influential cases) were verified visually and with diagnostic tools (performance package); marginal and conditional R^2^ values were calculated with MuMIn. Post hoc comparisons of estimated marginal means were performed with the emmeans package using Tukey adjustment for multiple comparisons. Perceived stress (PSS-10) pre–post changes were analysed using the Wilcoxon signed-rank test within each group. Between-group differences in the magnitude of change were examined with the Mann–Whitney U test on change scores (post–pre). Effect sizes for within-group changes were expressed as rank-biserial correlation (r) and for the between-group comparison as Cohen’s d. Cybersickness symptoms (VRSQ subscales) were analysed using the Friedman test across the five intervention days, with post hoc pairwise Wilcoxon signed-rank tests (Bonferroni-corrected) and Kendall’s W as the effect-size measure. The level of statistical significance was set at α = 0.05 (two-tailed). Sample size was determined for the primary physiological outcome, defined as the Group × Time interaction for lnRMSSD across the four within-session epochs. The calculation was based on the planned repeated-measures structure of this study, including two groups, four 5-minute epochs within each session, and five consecutive intervention days. Monte Carlo simulations were performed using the simr package in R to estimate the statistical power of the linear mixed-effect model. A total sample of 60 participants, allocated in a predefined 2:1 ratio to the immersive VR group (*n* = 40) and the non-immersive control group (*n* = 20), was estimated to provide approximately 80% power to detect a large Group × Time interaction corresponding to partial η^2^ ≥ 0.25. The assumption of a large effect size was considered appropriate because this study was designed as a proof-of-concept trial focused on immediate, short-term psychophysiological responses under highly standardized experimental conditions. The 2:1 allocation ratio was selected to increase the number of observations collected under immersive VR exposure, which was the main condition of interest, while maintaining an active non-immersive comparator group exposed to identical relaxation content. Perceived stress measured with the PSS-10 was treated as a key secondary psychological outcome; therefore, this study was not primarily powered to detect between-group differences in PSS-10 change scores. More broadly, the sample-size calculation was performed specifically for the primary physiological outcome, defined as the Group × Time interaction for lnRMSSD. This study was not powered separately for all secondary physiological, psychological, and tolerability outcomes. Accordingly, findings related to these secondary outcomes should be interpreted as supportive and exploratory rather than confirmatory. Because the intervention lasted only five consecutive days, was supervised, and involved brief 10-minute sessions, dropout was expected to be minimal and no additional inflation for attrition was applied. All randomized participants completed the intervention and were included in the final analysis.

## 3. Results

### 3.1. Heart Rate Variability Outcomes

Analysis of heart rate variability parameters using linear mixed-effect models revealed a statistically significant Group × Time interaction for all indices of autonomic nervous system activity (all *p* < 0.001; Table 2). The model accounted for the random effects of participants, including a random intercept and random slope, and included Day (1–5) as an additional repeated factor. Estimated marginal means (EMMeans ± SE) for the key HRV parameters across the four time points (T1–T4) are presented in Table 2, while the full results of the linear mixed-effect models are shown in Table 3.

In the immersive VR group, exposure to the virtual environment elicited rapid and pronounced activation of the parasympathetic nervous system. During the first half of the 10-minute session (T2), lnRMSSD increased significantly from 3.67 ± 0.05 at T1 to 3.88 ± 0.06 (*p* < 0.001), reaching its peak value of 3.93 ± 0.06 during the second half of the session (T3). This beneficial effect persisted after the end of exposure (T4: 3.87 ± 0.05). In parallel, mean heart rate decreased from 72.3 ± 1.3 bpm at T1 to 67.7 ± 1.4 bpm at T3, while the PNS index increased from 0.11 ± 0.07 to 0.91 ± 0.08. The Stress index decreased by nearly 1.3 units, from 4.85 ± 0.19 at T1 to 3.58 ± 0.20 at T3. A similar favourable pattern was observed for SDNN, which increased from 42.4 ± 2.2 ms at T1 to 54.0 ± 2.5 ms at T3, and for HF power, indicating strong and rapid modulation of autonomic balance toward parasympathetic dominance.

In the control group, in which the relaxation film was presented on a screen, the observed changes were weaker and less sustained. The increase in lnRMSSD was smaller and reached its maximum value of 3.76 ± 0.05 at T3. The decrease in mean heart rate and the increase in the PNS index were also smaller than in the immersive VR group at all time points after T1. The effect size of the Group × Time interaction was large for all parameters, with partial η^2^ values ranging from 0.32 to 0.43 (Table 2). Additional parameters, including pNN50, LF power, HF power, LF/HF ratio, and total spectral power, confirmed these differences and are presented in detail in the supplementary material (File S1).

**Figure 2:**
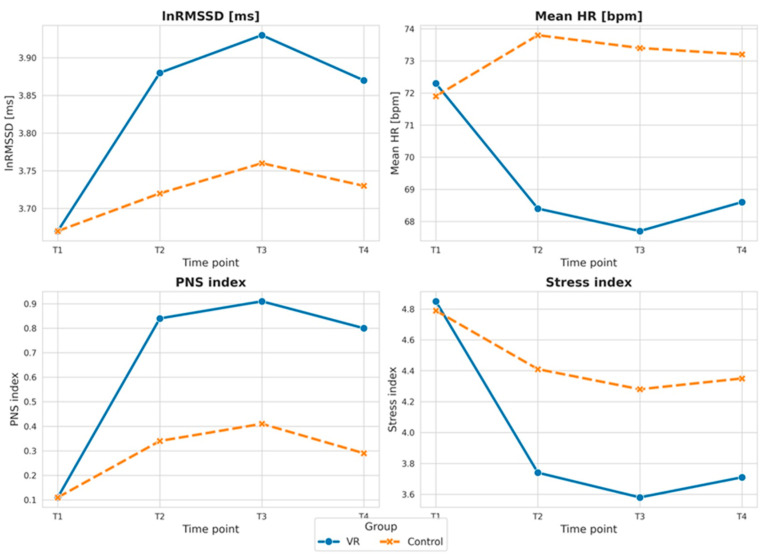
Dynamics of selected heart rate variability parameters across time points (T1–T4) in the immersive VR group and the non-immersive control group.

The dynamics of changes in selected HRV parameters over time are shown in Figure 2. The graph suggests a more pronounced short-term autonomic response in the immersive VR group compared with the non-immersive control group. In the immersive VR group, no significant differences were found between individual days of the intervention (all post hoc comparisons *p* > 0.05), indicating that the relaxation effect reached a plateau after the first sessions and remained highly reproducible across the five days. In the control group, no significant cumulative effect over time was observed, suggesting the limited effectiveness of the non-immersive form of relaxation.

### 3.2. Perceived Stress Outcomes

**Figure 3:**
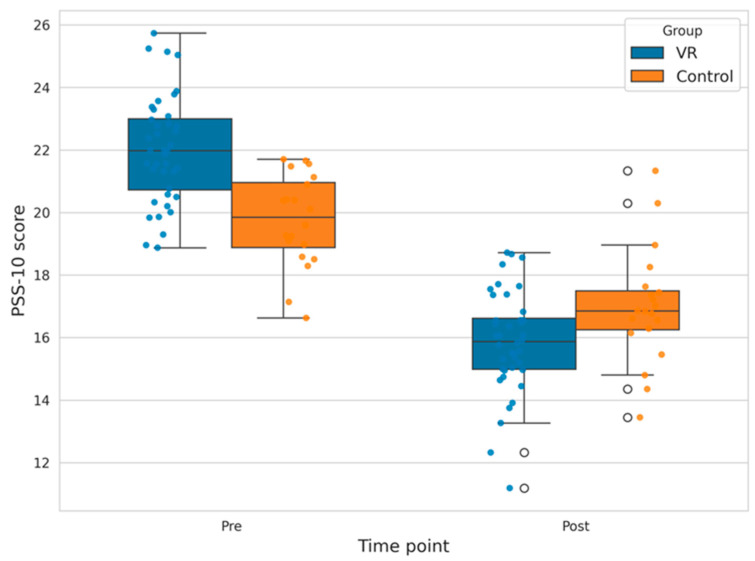
Changes in perceived stress (PSS-10 scores) from pre-intervention (day 1) to post-intervention (day 5) in the immersive virtual reality group and the non-immersive control group.

Perceived stress, measured using the Perceived Stress Scale-10 (PSS-10), decreased significantly in both groups. In the immersive VR group, scores decreased from a pre-intervention median of 22.0 [IQR: 19.0–26.0] to 16.0 [IQR: 13.0–20.0], with a mean reduction of 6.5 ± 4.2 points (Wilcoxon Z = −4.72, *p* < 0.001, r = 0.65). In the non-immersive control group, the corresponding reduction was smaller, from 20.5 [IQR: 16.0–25.0] to 15.5 [IQR: 13.0–22.3], with a mean reduction of 2.9 ±5.1 points (Z = −2.49, *p* = 0.013, r = 0.39). Between-group comparison of change scores revealed a significantly greater reduction in the immersive VR group (Mann–Whitney U = 248, *p* = 0.009, r = 0.37, Cohen’s d = 0.78), confirming that immersion produced a more pronounced stress-reducing effect than non-immersive exposure to the same content. Changes in PSS-10 scores from pre-intervention to post-intervention are shown in Figure 3.

### 3.3. Cybersickness Symptoms

Cybersickness symptoms were assessed only in the immersive VR group as a tolerability outcome related to exposure to the head-mounted virtual reality environment. Values for all three VRSQ subscales were low on the first day of the intervention and showed a decreasing trend across the five consecutive days. Mean values decreased from 1.27 ± 1.55 to 0.85 ± 1.12 for Nausea, from 3.58 ± 3.12 to 2.02 ± 2.30 for Oculomotor symptoms, and from 2.90 ± 2.81 to 1.81 ± 1.93 for Disorientation between day 1 and day 5, respectively. These findings suggest good short-term tolerability of repeated immersive VR exposure.

## 4. Discussion

The COVID-19 pandemic has led to a range of long-term psychological consequences in the student population, contributing to an increased prevalence of stress, anxiety, depressive symptoms, and reduced overall psychological well-being. The baseline PSS-10 scores indicated elevated perceived stress in the study sample, which is consistent with current evidence on the mental health status of students in the post-pandemic period. The present findings suggest that short-term immersive VR relaxation may be a feasible and well-tolerated experimental approach for inducing short-term psychophysiological changes in university students. However, its effectiveness, durability, and practical value in routine university settings require confirmation in larger randomized controlled trials with longer follow-up. The decrease in PSS-10 scores was more pronounced in the VR group and was accompanied by a larger effect size than that observed in the control condition. At the same time, the significant reduction in perceived stress in the non-immersive control group suggests that relaxation content itself may exert beneficial effects. However, the greater improvement observed in the immersive VR condition indicates that immersion may enhance the stress-reducing potential of relaxation-based interventions. Importantly, the immersive VR intervention not only elicited a large within-group reduction in perceived stress but also produced a statistically greater improvement than the non-immersive control condition (*p* = 0.009, Cohen’s d = 0.78). This finding underscores the added value of immersion beyond the relaxation content itself and is consistent with the notion that heightened presence and sensory engagement facilitate deeper disengagement from everyday stressors. This effect may be explained by the specific characteristics of immersive VR, including the sense of presence, attentional engagement, and temporary disengagement from external or everyday stressors. By creating a controlled, multisensory, and emotionally engaging environment, immersive VR may facilitate a deeper relaxation response than conventional screen-based exposure. Importantly, the subjective improvement in perceived stress was accompanied by favourable changes in autonomic nervous system activity, as reflected by HRV parameters. In the VR group, exposure to the virtual environment elicited a rapid increase in parasympathetic indices, including lnRMSSD, SDNN, and the PNS index, together with a decrease in mean heart rate, SNS index, and Stress index. These findings suggest a shift in autonomic balance toward parasympathetic predominance during immersive relaxation.

A particularly relevant finding was that the relaxation effect in the VR group appeared rapidly and remained highly reproducible across the five-day intervention. No significant differences were observed between individual days of the intervention, indicating that the autonomic relaxation response reached a plateau already after the first sessions rather than showing a progressive cumulative increase over time. This observation has practical relevance, as it suggests that even brief immersive VR relaxation sessions may be sufficient to induce a stable psychophysiological response. Such a pattern may support the implementation of short VR-based relaxation protocols in academic settings, where time constraints and limited access to psychological support are common barriers.

The findings are consistent with previous evidence indicating that the COVID-19 pandemic substantially affected the mental health of university students. In the meta-analysis by Li et al., which included 27 studies and more than 700,000 students, the prevalence of depressive symptoms, anxiety symptoms, and stress after the outbreak of the COVID-19 pandemic was estimated at 39%, 36%, and 33%, respectively [4]. Martins et al. emphasized that social isolation, restricted interpersonal contact, and academic overload were among the main factors associated with the deterioration of student mental health, contributing to the persistence of elevated psychological tension after the pandemic period [22]. Similar observations were reported by Le Vigouroux et al., who found that a substantial proportion of students continued to experience elevated stress symptoms and emotional difficulties during the post-pandemic period [23]. In this context, digital and immersive interventions may represent a promising direction for mental health support in young adults.

The present study also responds to an important gap identified in the literature. Previous studies on VR relaxation have often focused primarily on subjective psychological outcomes and general well-being indicators, whereas fewer studies have incorporated objective physiological measures such as HRV. Sailaa et al. highlighted that, although VR relaxation may be beneficial for student well-being, the available evidence remains heterogeneous with regard to intervention protocols, outcome measures, and physiological assessment [24]. By combining perceived stress assessment with HRV-derived autonomic indices, the present study provides a more comprehensive evaluation of the psychophysiological effects of immersive VR relaxation.

The observed autonomic changes are consistent with the theoretical assumption that relaxation-based VR interventions may modulate the stress response through parasympathetic activation. In the present study, immersive VR exposure was associated with a significant decrease in Stress index and sympathetic activity, expressed by the SNS index, together with an increase in parasympathetic activity, reflected by the PNS index. Improvements in HRV parameters associated with vagal modulation, particularly lnRMSSD and SDNN, further support the interpretation that immersive VR relaxation may enhance autonomic regulation. These findings suggest that VR-based relaxation may act not only at the subjective psychological level, but also through measurable physiological mechanisms involved in stress recovery.

The present observations are in line with previous studies reporting beneficial effects of VR-based interventions on stress and psychological distress. Riva et al. demonstrated that a 360° VR-based self-help intervention used during the COVID-19 lockdown reduced stress, depressive symptoms, and general psychological distress, suggesting that immersion in a safe and naturalistic virtual environment may help individuals distance themselves from everyday stressors [25]. Similarly, Kim et al. reported significant reductions in stress and anxiety following a VR relaxation intervention in individuals with high stress levels. Their study also showed favourable changes in physiological parameters, including an increase in NN50 and a decrease in the LF/HF ratio, which were interpreted as reflecting increased parasympathetic activity and improved autonomic regulation of the stress response [26]. The direction of these findings is consistent with the present results, in which increases in parasympathetic HRV indices were accompanied by reductions in stress-related autonomic markers.

VR technology has also been used as a stress-reduction tool among healthcare professionals working under pandemic-related pressure. Nijland et al. reported a 39.9% reduction in perceived stress after a single 10-minute immersive VR relaxation session among intensive care nurses [27]. Beverly et al. also demonstrated that a brief tranquil VR experience significantly reduced subjective stress among frontline healthcare workers during the COVID-19 pandemic, with the proportion of participants reporting high stress decreasing after the intervention [28]. Although these studies were conducted in healthcare professionals rather than students, their findings support the broader potential of short, immersive VR-based relaxation protocols as feasible stress-reduction tools in populations exposed to increased psychological demands.

In the post-pandemic period, VR has also gained attention in the context of telerehabilitation and home-based health support. Studies involving individuals with post-COVID conditions have suggested that VR-based rehabilitation programmes may improve exercise tolerance, daily activity, and functional status [29]. In addition, VR-based fitness and exergaming interventions have been discussed as strategies to support mental health and physical activity during periods of social restriction and reduced mobility [30,31]. These observations indicate that VR may have broader applicability beyond relaxation alone, including rehabilitation, behavioural activation, and support for psychological well-being. However, in the context of the present study, the most relevant application remains the use of short immersive relaxation sessions to reduce stress and modulate autonomic nervous system activity in students.

Despite the favourable psychophysiological effects observed in the present study, potential adverse effects related to VR exposure should also be considered. One of the most commonly described limitations of VR use is cybersickness, which may include dizziness, nausea, disorientation, and visual discomfort. Oh and Son indicated that the severity of cybersickness may depend on exposure duration, image quality, the type of movement within the virtual environment, and individual susceptibility [32]. Groenveld et al. also noted that most adverse symptoms associated with VR use are typically mild and transient [29]. In the present study, cybersickness symptoms in the VR group were low from the first day of the intervention and decreased across subsequent days. Because cybersickness was assessed only in the immersive VR group, the present findings should be interpreted as a within-group tolerability assessment of repeated immersive VR exposure rather than as a direct between-group safety comparison. This favourable tolerability profile may be related to the short duration of the sessions and the use of a relaxing, relatively static natural environment. Similar observations were reported by Anderson et al., who showed that short-term exposure to immersive natural scenes was well tolerated by participants [13].

The present study has several limitations that should be taken into account when interpreting the findings. First, although this study included a non-immersive control condition and objective HRV parameters, the intervention was short-term and did not include a follow-up assessment. Therefore, the durability of the observed psychological and physiological effects after completion of the intervention remains unknown. Second, perceived stress was assessed using a self-report measure, which may be influenced by expectancy effects and subjective interpretation of the intervention. Third, although HRV provides valuable information on autonomic nervous system regulation, this study did not include additional physiological markers, such as cortisol, electrodermal activity, or respiratory parameters, which could provide a broader picture of stress-response modulation. Because cybersickness was assessed only in the immersive VR group, direct comparisons of intervention tolerability between the immersive and non-immersive conditions were not possible. Moreover, participants were recruited from two universities in one Polish city, which limits the generalizability of the findings to broader student populations, other countries, and different academic settings. The statistically significant baseline age difference between groups should be considered an important limitation. Although the mean age differed by approximately three years, this disparity may still be relevant within the relatively narrow age range of the study population. The magnitude of its influence is difficult to determine, as even relatively modest age differences in young adults may be associated with variation in autonomic regulation, HRV parameters, perceived stress, and familiarity with immersive technologies. Therefore, the possibility that the baseline age imbalance contributed, at least partly, to the observed between-group differences cannot be excluded. Consequently, the findings should be interpreted cautiously, and the observed effects cannot be attributed solely to the immersive nature of the intervention. In addition, because the sample-size calculation was based on the primary physiological outcome, this study may have had insufficient statistical power for some secondary outcomes. Therefore, the findings related to the secondary physiological, psychological, and tolerability outcomes should be interpreted as supportive and exploratory rather than confirmatory. Finally, although complete post-intervention PSS-10 data were used for all participants, this study would benefit from a longer follow-up to assess the durability of the observed effects. Moreover, the intervention was conducted under highly controlled and supervised laboratory conditions, which may not fully reflect routine use in university settings. Under real-world conditions, participant adherence, environmental distractions, access to equipment, unsupervised use, and repeated long-term exposure may differ substantially from those observed in the present study. Future pragmatic trials should therefore evaluate the effectiveness, feasibility, and acceptability of immersive VR relaxation under routine academic conditions and include longer follow-up periods to determine whether the observed effects are maintained over time. Further research should also compare different types of virtual environments, session durations, and exposure frequencies to identify the optimal parameters of VR-based relaxation interventions. In addition, future trials should consider integrating subjective, behavioural, and physiological outcomes to clarify the mechanisms through which immersive VR influences stress regulation. Particular attention should also be given to the potential mediating role of autonomic nervous system activity in the relationship between VR exposure and perceived stress reduction.

Overall, the present findings suggest that immersive VR relaxation may induce short-term psychophysiological changes under controlled experimental conditions. However, the observed effects should be interpreted as preliminary and limited to the immediate post-intervention period. Larger randomized controlled trials with longer follow-up are needed before conclusions regarding durability, implementation, or practical use in university settings can be made. The beneficial effects observed in HRV parameters indicate that immersive relaxation environments may facilitate psychophysiological recovery by enhancing parasympathetic activity and reducing sympathetic stress-related activation. Brief VR-based relaxation sessions may represent a promising approach for supporting stress management in university students; however, larger randomized controlled trials with longer follow-up are needed before broader implementation can be recommended.

## 5. Conclusions

The present study suggests that a short-term immersive virtual reality relaxation intervention may contribute to short-term reductions in perceived stress and favourable modulation of autonomic nervous system activity in university students under controlled experimental conditions. Compared with non-immersive exposure to identical relaxation content, participants receiving immersive VR demonstrated greater short-term improvements in selected HRV parameters and perceived stress. However, these findings should be interpreted with caution due to the baseline age imbalance between groups, the short intervention period, the lack of long-term follow-up, and the possibility of expectancy or novelty effects associated with immersive technology. Because an age-adjusted sensitivity analysis was not performed, the extent to which the baseline age imbalance contributed to the observed between-group differences remains uncertain. Residual confounding by age therefore cannot be excluded. Consequently, no conclusions can be drawn regarding the durability of the observed effects or their effectiveness in routine university practice. Future randomized controlled trials with balanced groups, longer follow-up periods, and additional physiological and behavioural outcome measures are needed to confirm these preliminary findings and establish the clinical and practical relevance of immersive VR relaxation.

## Figures and Tables

**Table 1:** Baseline demographic and clinical characteristics of the study participants.

Characteristic	Immersive VR Group (*n* = 40)	Non-Immersive Control Group (*n* = 20)	*p*-Value
Age (years), mean ± SD	23.0 ± 0.82	19.95 ± 1.46	0.001
Female, *n* (%)	30 (75%)	12 (60%)	0.25
Baseline PSS-10, median [IQR]	22.0 [19.0–26.0]	20.5 [16.0–25.0]	0.31
Baseline lnRMSSD (T1), mean ± SE	3.67 ± 0.05	3.67 ± 0.04	0.98

**Table 2:** Estimated marginal means (±SE) of key heart rate variability parameters at four time points (T1–T4) by group.

Parameter	Group	T1	T2	T3	T4
Mean HR [bpm]	VR	72.3 ± 1.3	68.4 ± 1.4	67.7 ± 1.4	68.6 ± 1.3
	Control	71.9 ± 1.2	73.8 ± 1.3	73.4 ± 1.3	73.2 ± 1.2
lnRMSSD [ms]	VR	3.67 ± 0.05	3.88 ± 0.06	3.93 ± 0.06	3.87 ± 0.05
	Control	3.67 ± 0.04	3.72 ± 0.05	3.76 ± 0.05	3.73 ± 0.04
SDNN [ms]	VR	42.4 ± 2.2	51.6 ± 2.5	54.0 ± 2.5	50.7 ± 2.2
	Control	41.9 ± 2.1	44.7 ± 2.4	46.1 ± 2.4	45.3 ± 2.1
PNS index	VR	0.11 ± 0.07	0.84 ± 0.08	0.91 ± 0.08	0.80 ± 0.07
	Control	0.11 ± 0.06	0.34 ± 0.07	0.41 ± 0.07	0.29 ± 0.06
SNS index	VR	0.08 ± 0.06	−0.60 ± 0.07	−0.72 ± 0.07	−0.57 ± 0.06
	Control	0.09 ± 0.05	−0.19 ± 0.06	−0.28 ± 0.06	−0.22 ± 0.05
Stress index	VR	4.85 ± 0.19	3.74 ± 0.20	3.58 ± 0.20	3.71 ± 0.19
	Control	4.79 ± 0.18	4.41 ± 0.19	4.28 ± 0.19	4.35 ± 0.18

**Table 3:** Fixed effects for the Group × Time interaction from linear mixed-effect models predicting HRV parameters.

**HRV Parameter**	**F (df_1_, df_2_)**	***p*** **-Value**	**Partial η^2^**	**Standardized β**
lnRMSSD	27.85 (3, 228)	<0.001	0.43	0.66
Mean HR	19.42 (3, 228)	<0.001	0.34	−0.58
SDNN	17.61 (3, 228)	<0.001	0.32	0.55
PNS index	21.74 (3, 228)	<0.001	0.38	0.62
SNS index	20.39 (3, 228)	<0.001	0.37	−0.60
Stress index	24.81 (3, 228)	<0.001	0.40	−0.63

## Data Availability

The data presented in this study are available on request from the corresponding author. The data are not publicly available due to privacy restriction.

## Supplementary Materials

The following supporting information can be downloaded at: https://www.mdpi.com/article/10.3390/healthcare14152322/s1. Supplementary File S1: CONSORT 2025 checklist of information to include when reporting a randomised trial*.

## Abbreviations

COVID-19	Coronavirus Disease 2019
HRV	Heart Rate Variability
HF	High Frequency
IQR	Interquartile Range
LF	Low Frequency
LMM	Linear Mixed-Effect Model
lnRMSSD	Natural Logarithm of the Root Mean Square of Successive Differences
PNS	Parasympathetic Nervous System
PSS-10	Perceived Stress Scale-10
RR intervals	R-R Intervals
SD	Standard Deviation
SDNN	Standard Deviation of Normal-to-Normal Intervals
SE	Standard Error
SNS	Sympathetic Nervous System
VRSQ	Virtual Reality Sickness Questionnaire
VR	Virtual Reality
