# Supplementary material for: Short-Term Psychophysiological Effects of Immersive Versus Non-Immersive Relaxation in University Students: A Randomized Controlled Trial
Source: Healthcare (Basel). 2026 Aug 1;14(15):2322. doi: 10.3390/healthcare14152322 (PMC13465558; doi:10.3390/healthcare14152322)
Supplement: Supplementary file 1 [file healthcare-14-02322-s001.zip › healthcare-4384561-supplementary.pdf]

CONSORT 2025 checklist of information to include when reporting a randomised trial*

Section / Topic	No	CONSORT 2025 checklist item description	Reported on page no.
**Title and abstract**			
Title and structured abstract	1a	Identification as a randomised trial	Page 1, Title
	1b	Structured summary of the trial design, methods, results, and conclusions	Pages 1-2, Abstract
**Open science**			
Trial registration	2	Name of trial registry, identifying number (with URL) and date of registration	Pages 5 and 16. Registered at ClinicalTrials.gov, identifier NCT06480409. The registration URL and date of registration are not reported.
Protocol and statistical analysis plan	3	Where the trial protocol and statistical analysis plan can be accessed	Not publicly available; no separate trial protocol or statistical analysis plan was published.
Data sharing	4	Where and how the individual de-identified participant data (including data dictionary), statistical code and any other materials can be accessed	Page 17, Data

			Availability Statement. De-identified participant data are available from the corresponding author upon reasonable request. Statistical code, data dictionary, and other materials are not publicly available.
Funding and conflicts of interest	5a	Sources of funding and other support (e.g., supply of drugs), and role of funders in the design, conduct, analysis and reporting of the trial	Pages 16-17, Funding and Conflict of Interest Statements.
	5b	Financial and other conflicts of interest of the manuscript authors	Page 17, Conflict of Interest Statement
**Introduction**			
Background and rationale	6	Scientific background and rationale	Pages 2-3, Introduction
Objectives	7	Specific objectives related to benefits and harms	Pages 1 and 3, Abstract

			and Introduction, for the effectiveness objectives; Page 7, Measurements, for cybersickness assessment. A specific objective related to harms is not explicitly stated.
**Methods**			
Patient and public involvement	8	Details of patient or public involvement in the design, conduct and reporting of the trial	Not reported; no patient or public involvement.
Trial design	9	Description of trial design including type of trial (e.g., parallel group, crossover), allocation ratio, and framework (e.g., superiority, equivalence, non-inferiority, exploratory)	Page 4, Materials and Methods: randomized controlled trial with repeated measures, parallel-

			group design, and a 2:1 allocation ratio. The trial framework is not explicitly identified.
Changes to trial protocol	10	Important changes to the trial after it commenced including any outcomes or analyses that were not prespecified, with reason	Not applicable
Trial setting	11	Settings (e.g., community, hospital) and locations (e.g., countries, sites) where the trial was conducted	Page 4, Materials and Methods - university students from two universities in Opole, Poland.
Eligibility criteria	12a	Eligibility criteria for participants	Page 5, Qualification criteria.
	12b	If applicable, eligibility criteria for sites and for individuals delivering the interventions (e.g., surgeons, physiotherapists)	Not applicable
Intervention and comparator	13	Intervention and comparator with sufficient details to allow replication. If relevant, where additional materials describing the intervention and comparator (e.g., intervention manual) can be accessed	Pages 5-6, Intervention; Page 6, Figure 1.
Outcomes	14	Pre-specified primary and secondary outcomes, including the specific measurement variable (e.g., systolic blood pressure), analysis metric (e.g., change from baseline, final value, time to event), method of aggregation (e.g., median, proportion), and time point for each outcome	Pages 6-8, Measureme nts and

			Statistical analysis.
Harms	15	How harms were defined and assessed (e.g., systematically, non-systematically)	Pages 6–7, Measurements; Page 12, Cybersickness Symptoms
Sample size	16a	How sample size was determined, including all assumptions supporting the sample size calculation	Page 8, Statistical analysis.
	16b	Explanation of any interim analyses and stopping guidelines	Not applicable - no interim analyses or stopping guidelines.
Randomisation:			
Sequence generation	17a	Who generated the random allocation sequence and the method used	Page 4, Materials and Methods.
	17b	Type of randomisation and details of any restriction (e.g., stratification, blocking and block size)	Page 4, Materials and Methods - simple randomisation, 2:1 ratio.
Allocation concealment mechanism	18	Mechanism used to implement the random allocation sequence (e.g., central computer/telephone; sequentially numbered, opaque, sealed containers), describing any steps to conceal the sequence until interventions were assigned	Page 4, Materials and Methods.

Implementation	19	Whether the personnel who enrolled and those who assigned participants to the interventions had access to the random allocation sequence	Not fully reported.
Blinding	20a	Who was blinded after assignment to interventions (e.g., participants, care providers, outcome assessors, data analysts)	Page 4, Materials and Methods - participants and assessors were not blinded; analyses used anonymised data.
	20b	If blinded, how blinding was achieved and description of the similarity of interventions	Not applicable.
Statistical methods	21a	Statistical methods used to compare groups for primary and secondary outcomes, including harms	Pages 7-8, Statistical analysis.
	21b	Definition of who is included in each analysis (e.g., all randomised participants), and in which group	Page 8, Statistical analysis - all randomised participants were included.
	21c	How missing data were handled in the analysis	Not applicable - all randomised participants completed

			the intervention.
	21d	Methods for any additional analyses (e.g., subgroup and sensitivity analyses), distinguishing prespecified from post-hoc	Page 8, Statistical analysis - secondary outcomes were exploratory; no subgroup or sensitivity analyses.
**Results**			
Participant flow, including flow diagram	22a	For each group, the numbers of participants who were randomly assigned, received intended intervention, and were analysed for the primary outcome	Page 6, Figure 1.
	22b	For each group, losses and exclusions after randomisation, together with reasons	Page 6, Figure 1.
Recruitment	23a	Dates defining the periods of recruitment and follow-up for outcomes of benefits and harms	Not reported.
	23b	If relevant, why the trial ended or was stopped	Not applicable.
Intervention and comparator delivery	24a	Intervention and comparator as they were actually administered (e.g., where appropriate, who delivered the intervention/comparator, how participants adhered, whether they were delivered as intended [fidelity])	Pages 5-6, Intervention; Page 6, Figure 1.
	24b	Concomitant care received during the trial for each group	Not applicable.
Baseline data	25	A table showing baseline demographic and clinical characteristics for each group	Page 4, Table 1.
Numbers analysed, outcomes and estimation	26	For each primary and secondary outcome, by group:<br>● the number of participants included in the analysis<br>● the number of participants with available data at the outcome time point	Pages 8-12, Results, Tables 2-3

		• result for each group, and the estimated effect size and its precision (such as 95% confidence interval)<br>• for binary outcomes, presentation of both absolute and relative effect size	and Figures 2-3.
Harms	27	All harms or unintended events in each group	Page 12, Cybersickness Symptoms; Pages 14-15, Discussion.
Ancillary analyses	28	Any other analyses performed, including subgroup and sensitivity analyses, distinguishing pre-specified from post-hoc	Pages 8 and 10, Statistical analysis and post-hoc analyses; no subgroup or sensitivity analyses.
**Discussion**			
Interpretation	29	Interpretation consistent with results, balancing benefits and harms, and considering other relevant evidence	Pages 12-16, Discussion and Conclusions.
Limitations	30	Trial limitations, addressing sources of potential bias, imprecision, generalisability, and, if relevant, multiplicity of analyses	Page 15, Limitations; Page 16, Conclusions.

*We strongly recommend reading this statement in conjunction with the CONSORT 2025 Explanation and Elaboration and/or the CONSORT 2025 Expanded Checklist for important clarifications on all the items. We also recommend reading relevant CONSORT extensions. See www.consort-spirit.org.

Citation: Hopewell S, Chan AW, Collins GS, Hróbjartsson A, Moher D, Schulz KF, et al. CONSORT 2025 Statement: updated guideline for reporting randomised trials. BMJ. 2025; 388:e081123. https://dx.doi.org/10.1136/bmj-2024-081123.

© 2025 Hopewell et al. This is an Open Access article distributed under the terms of the Creative Commons Attribution License (https://creativecommons.org/licenses/by/4.0/), which permits unrestricted use, distribution, and reproduction in any medium, provided the original work is properly cited.
